# Patterns and correlates of objectively measured free-living physical activity in adults in rural and urban Cameroon

**DOI:** 10.1136/jech-2014-205154

**Published:** 2015-04-04

**Authors:** Felix Assah, Jean Claude Mbanya, Ulf Ekelund, Nicholas Wareham, Soren Brage

**Affiliations:** 1 Department of Public Health, Faculty of Medicine and Biomedical Sciences, University of Yaoundé I, Yaoundé, Cameroon; 2 MRC Epidemiology Unit, University of Cambridge, Cambridge, UK

**Keywords:** PHYSICAL ACTIVITY, Epidemiology of chronic non communicable diseases, DEVELOPING COUNTR

## Abstract

**Background:**

Urbanisation in sub-Saharan Africa is changing lifestyles and raising non-communicable disease burden. Understanding the underlying pattern of physical activity and its correlates may inform preventive interventions. We examined correlates of objectively-measured physical activity in rural and urban Cameroon.

**Methods:**

Participants were 544 adults resident in rural (W-156, M-89) or urban (W-189, M-110) regions. Physical activity was measured using individually-calibrated combined heart rate and movement sensing over seven continuous days. Sociodemographic data were collected by self-report. Independent associations of sociodemographic correlates with physical activity energy expenditure (PAEE) or moderate-to-vigorous physical activity (MVPA) were analysed in multivariate regression models.

**Results:**

Rural dwellers were significantly more active than their urban counterparts (PAEE: 58.0 vs 42.9 kJ/kg/day; MVPA: 107 vs 62 min/day; MVPA of 150 min/week in >10 min bouts: 62 vs 39%) and less sedentary (923 vs 1026 min/day); p<0.001. There was no significant seasonal difference (dry vs rainy) in activity in urban dwellers whereas in rural dwellers activity was higher during dry seasons compared to rainy seasons (p<0.001). Age, obesity and education showed significant inverse associations with activity. Urban dwellers who considered themselves adequately active were only as active as rural dwellers who thought they were not adequately active.

**Conclusions:**

This is the first study providing data on sociodemographic patterning of objectively-measured physical activity in rural and urban sub-Saharan Africa. Age, urban residence, obesity and higher educational level are important correlates of lower levels of physical activity. These suggest targets for public health interventions to improve physical activity in Cameroon.

## Introduction

Non-communicable diseases (NCDs) are now the leading causes of death and disability in the world.^1^ In developing countries this burden of NCDs adds to a persistent high burden of infections; constituting a double disease burden despite limited healthcare resources. Physical inactivity is one of the four common risk factors of this NCD burden, together with unhealthy diet, smoking and excessive consumption of alcohol. Population physical activity levels have been reported to be declining in developed and developing countries.^2–4^ The decline in developing countries may be due to urbanisation as demonstrated by a rural-to-urban negative gradient of physical activity levels that have been reported in many studies using self-report of activity,^5^ ^6^ and as previously reported in this cohort using objective measurement of free-living activity.^7^ There is strong evidence that physical activity confers numerous health benefits including reducing morbidity and mortality from cardiovascular diseases and cancers.^8–10^ Consequently, recommendations on physical activity for promoting and maintaining a healthy life have been proposed.^11^ ^12^

Physical activity is a complex behaviour that involves an interaction between personal, interpersonal, policy and environmental factors.^13^ ^14^ Therefore, the design and implementation of appropriate public health interventions for physical activity promotion is a daunting task. Accurate and objective measurement of free-living physical activity and its correlates would enable targeted and quantifiable interventions. The current literature on physical activity in sub-Saharan Africa has mostly used self-reported assessment of physical activity,^5^ ^6^ ^15–17^ which is limited by recall bias and also restricted in the dimensions and domains of activity measured. We carried out this study to describe patterns of objectively-measured free-living physical activity energy expenditure (PAEE) and its underlying intensity distribution as time spent sedentary and in moderate-to-vigorous physical activity (MVPA) in rural and urban Cameroon, and to examine some anthropometric and sociodemographic correlates of activity in this population.

## Methods

The methods used in this study have been reported previously.^7^

### Participants and setting

Participants for this study were 25–55 years old and recruited from two urban areas (Yaoundé—the capital city of the Centre region and of Cameroon, and Bamenda—the capital city of the North Western region) and two rural areas (Mbankomo and Bafut) in these same regions. A total of 651 volunteers (urban: N=348, mean age=37.9±9.1 years; rural: N=303, mean age=38.5±8.3 years) took part in this study. Volunteers were provided detailed information about the study and invited to attend a testing session at their local hospital. Volunteers who had conditions that did not allow them to take part in exercise testing according to study procedures were excluded.

Ethical approval for the study was obtained from the Cameroon National Ethics Committee. All participants provided signed informed consent.

### Sociodemographic data

An interviewer-administered questionnaire was completed by all participants with sociodemographic variables recorded as reported. Smoking and alcohol drinking was recorded as ‘Yes’ or ‘No’, with ex-smokers or ex-drinkers classified as ‘No’. Data on frequency of drinking in four categories (<1 day/month, 1–3 days/month, 1–4 days/week and >5 days/week) were also collected. Data on frequency of fruit and vegetable consumption was collected as times per day and/or times per week.

Occupational status was collected in four categories: employed, self-employed, non-paid work or unemployed/retired. Information was also collected on habitual occupational activity intensity in three categories with prompting from the interviewer:

- Light; involving mainly sitting activities or standing without additional effort, light cleaning, ironing, cooking, driving
- Moderate; continuous walking, intense cleaning, sweeping, brushing, gardening, light load carriage, painting, etc
- Intense; carrying heavy loads, construction work, farming, wood splitting, other similarly intense activities, etc.

### Physical activity measurement

Free-living physical activity was measured using a combined heart rate and movement sensor (Actiheart, Cambridge Neurotechnology Ltd, Papworth, UK); with individual calibration of the heart rate response to workload derived from a step test.^18^ ^19^ This test also provides an estimate of cardiorespiratory fitness (VO_2_max). Free-living physical activity data was recorded continuously in 1 min epochs over seven continuous days. The participants were requested to carry on with their normal habitual lifestyle, and wear the monitor at all times except for showering, bathing or activities like swimming. The minute-by-minute heart rate data was pre-processed,^20^ calibrated using the step test response, and combined with acceleration in a branched equation model^21^ to calculate physical activity intensity time-series during free-living. From these data, average PAEE and distribution of intensity were summarised, while minimising diurnal bias. Participants with at least 48 h of wear time were included in the analyses. The distribution of intensity of activity was expressed as average daily time spent at 19 different multiples of 1 MET, and summarised in broad categories as time spent sedentary (<1.5 MET), time spent in light intensity physical activity (LPA, 1.5–3 MET), and time spent in MVPA (>3 MET) in bouts from 1 to 10 min; 1 MET is notionally the energy cost of the resting state of an individual; for the present analyses, we used the standard value of 3.5 mL O_2_/min/kg (∼71 J/min/kg) as multiplier.^22^ Adherence to current physical activity guidelines^12^ was defined as accumulating at least 150 min/week of MVPA in bouts of at least 10 min duration.

Participants were also asked, without any prompting from the interviewer, to rate as “adequate” or “not adequate” their self-perceived level of habitual physical activity.

### Anthropometry and clinical measures

Height without shoes was measured using a standard rigid stadiometer. Waist and hip circumferences were measured to the nearest 0.1 cm using a D-loop non-stretch fibreglass tape. Body weight was measured using electronic clinical scales and body composition using bioimpedance (Tanita TBF-531 scales, Tanita UK Ltd, Uxbridge, Middlesex, UK). Body mass index (BMI) was categorised as normal: <25; overweight: ≥25 and <30; obese: ≥30 kg/m^2^.

### Calculations and statistical analyses

Analyses were carried out using STATA V.13.1 (StataCorp, College Station, Texas, USA). Where applicable, the level of statistical significance was considered at p<0.05.

**Figure 1:**
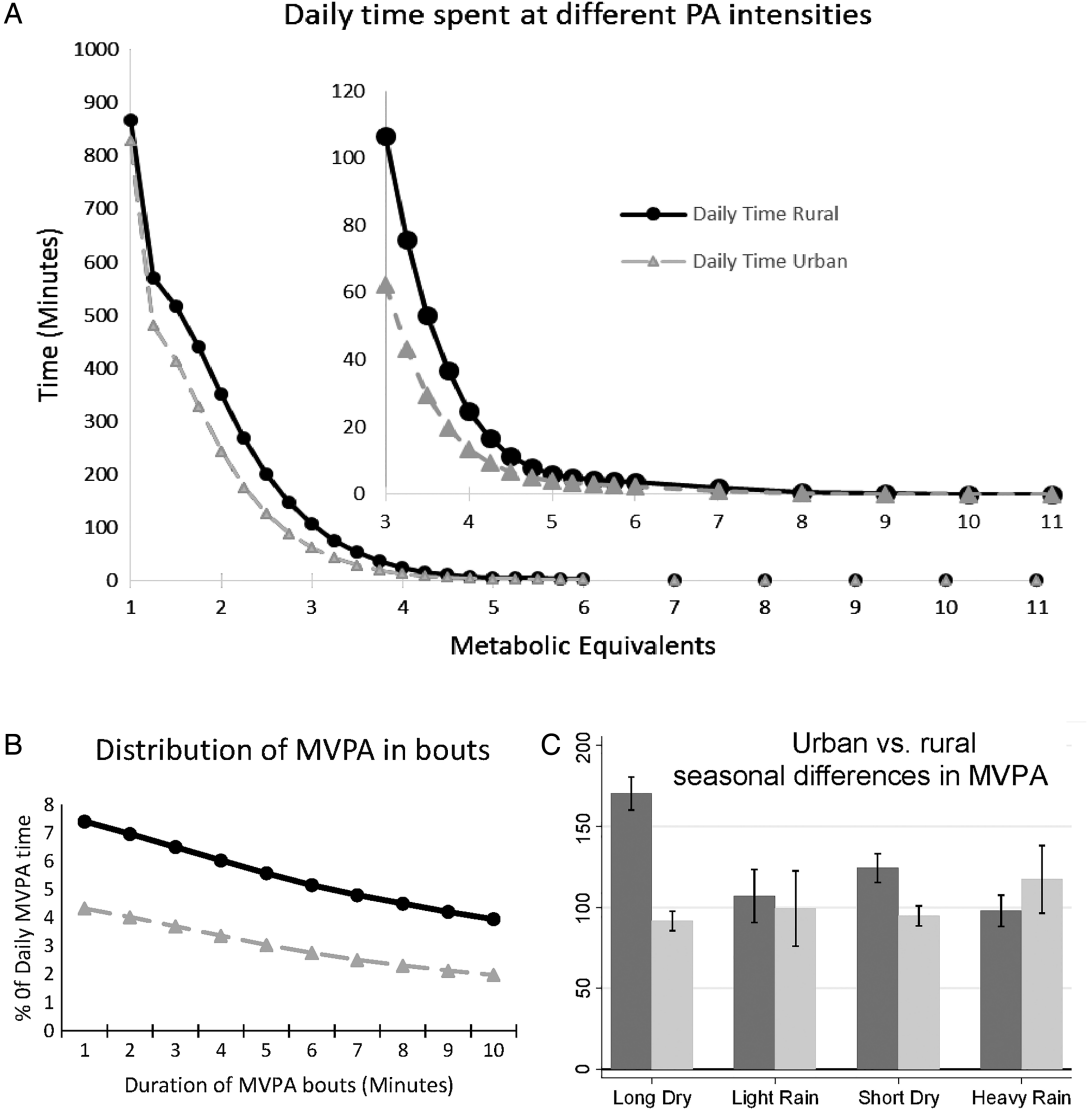
Distribution (A and B) and seasonal differences (C) of physical activity intensities between rural and urban dwellers in Cameroon (N=544). Intensity distributions (panel A) are significantly different between urban and rural dwellers (Wilk's λ, p<0.001), as is the distribution of moderate-to-vigorous physical activity (MVPA) in bouts (panel B, Wilk's λ, p<0.001). Dark bars/lines=Rural, Light bars/lines=Urban. Bars are means and error bars are SEM. Seasons are: Long Dry, December–May; Light Rain, June; Short Dry, July–September; Heavy Rain, October–November.

Descriptive characteristics of the study sample are presented stratified by rural/urban residential area and by sex. Student t tests or χ^2^ tests were used to assess differences in means or proportions, respectively. Multivariate test (Wilk's λ) was used to test differences in means of intensity and bout distributions of physical activity by urban/rural residential area (figure 1).

Associations between sociodemographic and anthropometric variables and PAEE or MVPA were examined in multivariate linear regression models. Multivariate logistic regression was used to assess correlates of meeting/not meeting current activity guidelines. Regression models are presented separately for rural and urban dwellers since our a priori aim was to understand correlates of physical activity in each setting. The regression models therefore explored differences in the dependent variable across levels or groups of independent variables, adjusted for age and sex, within the same residence.

## Results

A total of 544 individuals (199 men and 345 women, or 245 rural and 299 urban dwellers) with valid physical activity data are included in these analyses out of 651 volunteers who effectively participated. The mean (SD) duration of monitor wear was 6.4 (1.5) days; more than 95% of participants wore monitor for at least 3.5 days (see supplementary figure S1). Table 1 presents clinical and physical activity characteristics of the study sample, stratified by sex and residential site. Smoking was almost entirely a male habit, with 23% of men smoking compared to 1% of women. Obesity (BMI ≥30 kg/m^2^) was significantly more prevalent in women than men (32% vs 7%). Compared to rural dwellers, urban dwellers had a significantly higher prevalence of obesity (11% vs 32%) and also a higher fraction of body fat.

**Table 1:** Clinical and physical activity characteristics in rural and urban men and women (N=544)

	Women (n=345)			Men (n=199)		
	Rural (156)	Urban (189)	p Value	Rural (89)	Urban (110)	p Value
Age (years)	39.8±8.0	38.4±8.9	0.1	38.0±8.6	37.2±9.1	0.5
Education (years)*	8.1±4.1	11.9±5.4	<0.001	8.6±4.4	12.8±4.9	<0.001
BMI (kg/m^2^)*	24.8±4.7	28.7±5.3	<0.001	23.2±3.3	25.3±3.9	<0.001
WC (cm)*	84.5±10.3	93.4±12.2	<0.001	82.5±8.2	89.7±10.7	<0.001
Body fat (%)*	30.0±8.3	36.9±7.6	<0.001	16.1±5.4	20.2±6.6	<0.001
Obesity (BMI)*	23 (15%)	86 (46%)	<0.001	3 (3%)	11 (10%)	0.07
Central obesity (WC)*	102 (65%)	159 (84%)	<0.001	8 (9%)	30 (27%)	0.001
Smoking	1 (1%)	1 (1%)	0.9	18 (20%)	28 (25%)	0.4
Alcohol drinking	121 (78%)	140 (74%)	0.5	73 (82%)	95 (86%)	0.4
PAEE (kJ/kg/day)*	54.3±21.2	37.9±16.9	<0.001	64.6±25.0	51.5±22.9	<0.001
PAL*	1.79±0.26	1.63±0.23	<0.001	1.84±0.27	1.72±0.26	0.001
Sedentary time (min/day)*	943±147	1057±144	<0.001	889±148	973±156	<0.001
LPA (min/day)*	404±105	336±116	<0.001	421±100	377±117	0.007
MVPA (min/day)*	93±71	47±46	<0.001	131±98	89±75	0.001
MVPA, 10 min bouts (min/day)*	49±50	21±30	<0.001	70±70	41±48	0.001
VO_2_max (mL/kg/min)*	30.4±6.0	26.4±4.6	<0.001	37.8±8.3	37.3±7.7	0.6

Data are mean±SD or n (%). p Values test for difference in means using the t test or difference in proportion using the χ^2^ test.

*p<0.05 for combined rural versus urban difference.

BMI, body mass index; LPA, light intensity physical activity; MVPA, moderate-to-vigorous physical activity; PAEE, physical activity energy expenditure; PAL, physical activity level; WC, waist circumference.

The rural participants spent on average almost 35% more energy being physically active compared to the urban dwellers (Mean±SD: 58±23.2 vs 42.9±20.4 kJ/kg/day, p<0.001). This significant difference persisted after adjustment for age, sex, smoking, alcohol consumption, fruit and vegetable intake, body fat and education level. The rural dwellers spent significantly less time sedentary (difference of 103 min/day), and more time in MVPA (44 min/day) compared to urban dwellers (p<0.001). In rural and urban dwellers, men had significantly higher PAEE compared to women. Men spent significantly less time being sedentary and more time in LPA and MVPA.

Rural dwellers compared to their urban counterparts spent more time in physical activities of all intensities, and also at all minimum durations of bouts of MVPA (figure 1A, B); 62% of rural and 39% of urban dwellers accumulated at least 150 min/week of MVPA in bouts of >10 min (p<0.001). There was no difference in MVPA over the seasons in the urban dwellers (p=0.2); figure 1C. Meanwhile, in rural dwellers, MVPA (in bouts of >10 min or overall MVPA) was higher in the dry than in the rainy seasons (p<0.001). This seasonal pattern of MVPA in the rural and urban dwellers was similar to that of PAEE, but the reverse of sedentary time.

In multivariate linear models adjusted for age and sex, obesity was strongly inversely associated with PAEE in rural and urban dwellers; p<0.05 (table 2).

**Table 2:** **A**ssociations between sociopersonal characteristics and PAEE in rural and urban adult Cameroonians (N=544)

** **						
Correlates	PAEE (kJ/kg/day)					
	Rural (N=245)			Urban (N=299)		
	β	SE	p Value	β	SE	p Value
*Demographic and anthropometric*						
Age (years)	−0.42	0.18	0.02	−0.56	0.12	<0.001
BMI (kg/m^2^)	−1.40	0.33	<0.001	−0.93	0.22	<0.001
Normal	–			–		
Overweight	−10.0	3.29	0.003	−4.17	2.72	0.1
Obese	−19.43	4.68	<0.001	−9.88	2.81	0.001
WC (cm)	−0.55	0.15	<0.001	−0.44	0.1	<0.001
Normal	–			–		
Abdominal obesity	−5.52	3.47	0.1	−8.67	2.85	0.003
*Related lifestyle behaviours*						
Smoking	11.22	5.73	0.05	8.34	3.98	0.04
Alcohol drinking	5.65	3.53	0.1	5.89	2.64	0.03
Drinking frequency						
<1 day/month	–			–		
1–3 days/month	2.30	5.09	0.7	9.48	3.41	0.006
1–4 days/week	7.39	5.33	0.2	3.02	3.64	0.4
>5 days/week	13.42	6.01	0.03	−3.93	4.87	0.5
Fruits and vegetables						
<3 times/week	–			–		
3–6 times/week	9.19	3.44	0.008	3.81	2.32	0.1
≥7 times/week	10.54	4.10	0.01	7.77	3.32	0.02
*Social correlates*						
Marital status						
Single	–			–		
Married	11.98	3.85	0.002	3.0	2.9	0.3
Family size (>15 years)						
1–2	–			–		
3–5	−2.38	3.36	0.5	0.51	2.8	0.9
Over 5	−4.44	4.54	0.3	−6.05	3.21	0.06
School duration (years)	−0.77	0.36	0.03	−1.03	0.2	<0.001
Level of education						
<Primary school	–			–		
Primary school	1.02	3.59	0.8	−6.02	3.75	0.1
Secondary school	−7.23	4.93	0.1	−11.1	3.8	0.004
University	−10.44	9.56	0.3	−17.11	4.09	<0.001
Occupational status						
Employed	–			–		
Self-employed	11.20	3.96	0.005	8.94	2.82	0.002
Non-paid work	22.11	3.79	<0.001	8.01	2.74	0.004
Unemployed/retired	9.16	5.71	0.1	1.93	3.94	0.6
Occupational category						
Light activity	–			–		
Moderate activity	11.36	4.07	0.006	−1.56	2.83	0.6
Intense activity	19.17	3.05	<0.001	17.4	3.08	<0.001

Data are regression coefficients, which represent the difference in PAEE (kJ/kg/day) for a unit difference in exposure; stratified by rural/urban residential site. Estimates are adjusted for age and sex.

BMI, body mass index; PAEE, physical activity energy expenditure; WC, waist circumference.

Alcohol drinking and smoking were positively associated with PAEE, though of borderline statistical significance. Education (years spent in school) was significantly inversely associated with PAEE in urban and rural dwellers. People who did non-paid work or were self-employed had significantly higher PAEE than their counterparts who were employed. In the rural but not urban areas, married people had significantly higher PAEE than their single counterparts. The rural dwellers who reported their occupational activity to be moderate or intense had significantly higher PAEE compared to those in light occupations. Among urban dwellers, only those in intense occupations had significantly higher PAEE than any of the other occupational categories.

We also examined whether the associations between these anthropometric and sociodemographic correlates with MVPA were different from those observed for PAEE (table 3). Overall, a similar pattern of associations was observed with time spent in MVPA as was observed for PAEE (table 2). However, the associations between obesity variables and time spent in MVPA were stronger in rural than urban dwellers. Logistic regression models showed similar association between these correlates and meeting current physical activity guidelines (see online supplementary table S1).

**Table 3:** Associations between sociopersonal characteristics and time spent in MVPA in rural and urban adult Cameroonians (N=544)

** **						
Correlates	MVPA (min/day)					
	Rural (N=245)			Urban (N=299)		
	β	SE	p Value	β	SE	p Value
*Demographic and anthropometric*						
Age (years)	−0.94	0.64	0.1	−1.73	0.37	<0.001
BMI (kg/m^2^)	−5.79	1.18	<0.001	−2.69	0.68	<0.001
Normal	–			–		
Overweight	−47.04	11.82	<0.001	−14.02	8.41	0.1
Obese	−71.24	16.79	<0.001	−27.98	8.6	0.001
WC (cm)	−2.25	0.54	<0.001	−1.26	0.3	<0.001
Normal	–			–		
Abdominal obesity	−27.4	12.52	0.03	−29.32	8.64	0.001
*Related lifestyle behaviours*						
Smoking	48.9	20.7	0.02	26.65	12.14	0.03
Alcohol drinking	21.3	12.81	0.1	15.69	8.1	0.05
Drinking frequency						
<1 day/month	–			–		
1–3 days/month	9.42	19.04	0.6	20.35	10.88	0.06
1–4 days/week	25.14	19.91	0.2	−0.23	11.58	1
>5 days/week	41.29	22.43	0.07	−12.47	15.63	0.4
Fruits and vegetables						
<3 times/week	–			–		
3–6 times/week	39.28	12.38	0.002	13.94	7.14	0.05
≥7 times/week	46.59	14.77	0.002	18.22	10.13	0.07
*Social correlates*						
Marital status						
Single	–			–		
Married	30.25	14.11	0.03	6.49	8.95	0.5
Family size (>15 years)						
1–2	–			–		
3–5	−9.22	12.21	0.5	0.42	8.67	1
Over 5	−13.64	16.49	0.4	−16.91	9.94	0.09
School duration (years)	−2.44	1.32	0.07	−3.23	0.62	<0.001
Level of education						
<Primary school	–			–		
Primary school	1.8	13.13	0.9	−23.45	11.34	0.04
Secondary school	−20.96	17.98	0.2	−42.13	11.42	<0.001
University	−43.0	34.86	0.2	−58.59	12.3	<0.001
Occupational status						
Employed	–			–		
Self-employed	34.2	14.36	0.02	14.71	8.74	0.09
Non-paid work	79.2	13.77	<0.001	26.16	8.46	0.002
Unemployed/retired	34.32	20.7	0.1	2.63	12.07	0.8
Occupational category						
Light activity	–			–		
Moderate activity	35.57	14.73	0.02	−8.09	8.68	0.4
Intense activity	70.24	11.05	<0.001	52.62	9.35	<0.001

Data are regression coefficients, which represent the difference in MVPA (min/day) for a unit difference in exposure. Estimates are adjusted for age and sex.

BMI, body mass index; MVPA, moderate-to-vigorous physical activity; WC, waist circumference.

**Figure 2:**
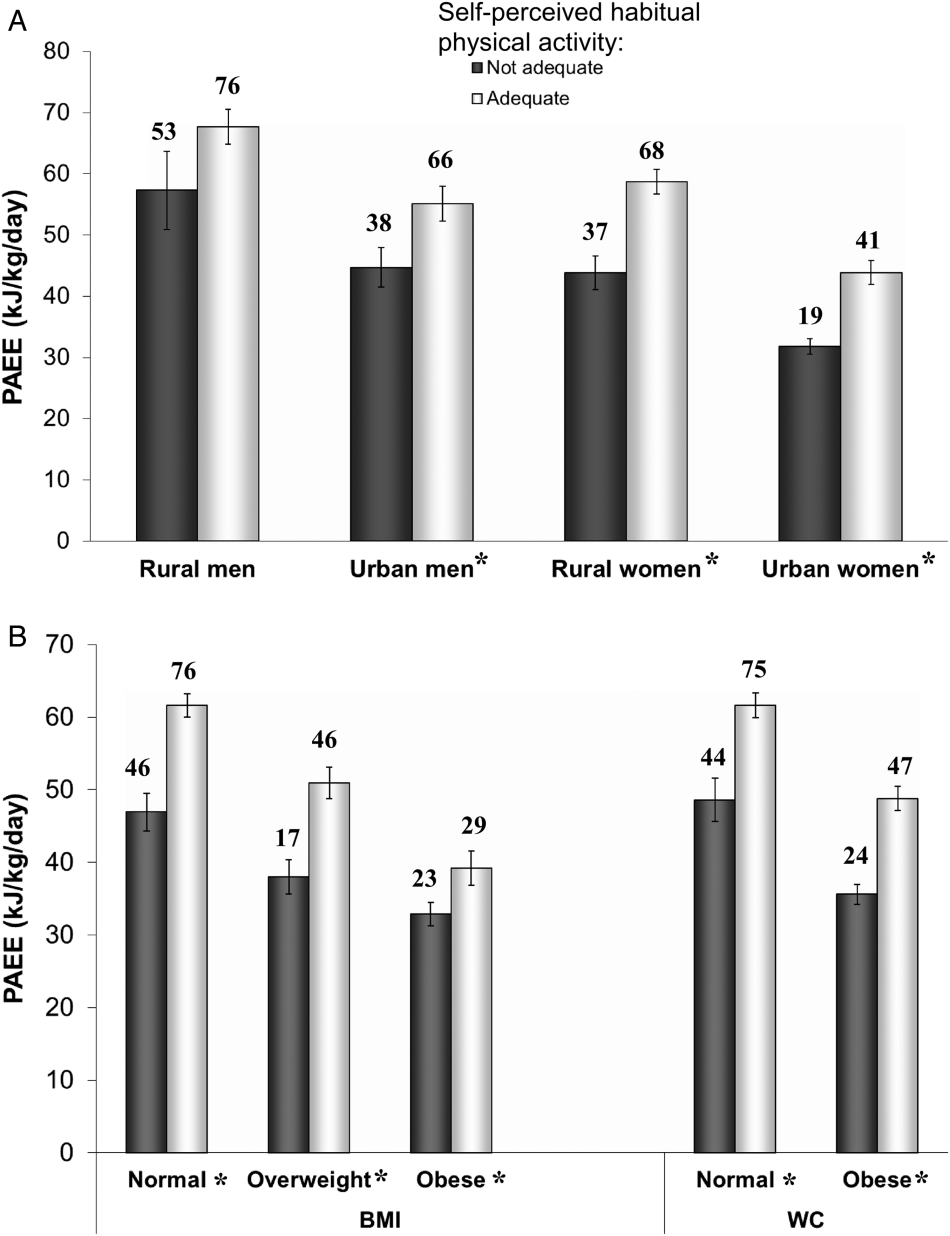
Comparison of objectively assessed physical activity energy expenditure by self-perceived adequacy of habitual physical activity in (A) rural and urban dwellers, and (B) by obesity categories. Bars are means and error bars are SEM. Numbers above bars are percentage meeting current physical activity guidelines. *p<0.05 for difference in mean PAEE between ‘not adequate’ and ‘adequate’ groups. BMI, body mass index; PAEE, physical activity energy expenditure; WC, waist circumference.

Finally participants were asked to rate their perceived level of habitual physical activity—stating whether it was “adequate” or “not adequate”. Figure 2A compares PAEE across categories of self-perceived adequacy of habitual physical activity by sex and residential site. Mean PAEE was significantly higher in men who thought their physical activity level was adequate compared to those who said it was not adequate (61.5±24.2 vs 48.9±24 kJ/kg/day, p=0.001). A similar trend was seen in women (52.2±20.7 vs 36.5±16.8 kJ/kg/day, p<0.001). Urban men and women who thought their physical activity level was adequate had similar PAEE as their rural counterparts who said their level was not adequate. When examining obesity and perception of habitual physical activity (figure 2B), mean PAEE was lower in obese people who said they were adequately active compared to people with normal weight who said their habitual activity was not adequate. Thirty per cent of people who said they were not adequately active met current physical activity guidelines while 38% of those who said they were sufficiently active did not meet same guidelines. Female sex and urban residence contributed more to this misclassification of self-perceived adequacy of activity level versus meeting current physical activity guidelines than male sex or rural residence.

## Discussion

In this population-based study of objectively-measured physical activity in free-living adults in sub-Saharan Africa, we report significant differences in sociodemographic correlates and patterns of physical activity between rural and urban dwellers. The rural dwellers were significantly more active than their urban counterparts; having a higher overall level of physical activity (PAEE and PAL), and spending significantly more time in MVPA but less time asleep or sedentary. Obesity was strongly inversely associated with PAEE in rural and urban dwellers.

Urbanisation in Africa has been suggested to be associated with lower levels of physical activity.^5^ ^23^ Conversely, in many developed societies, the reverse is true.^24^ ^25^ Almost all of the previous population-based studies of physical activity levels in sub-Saharan Africa have used subjective self-report methods.^2^ ^5^ ^6^ ^16^ ^26^ Self-reported levels of physical activity are limited by recall bias and differential misreporting.^27^ ^28^ Christensen *et al*^29^ employed objective measurement of physical activity in their study in Kenya. However, under-sampling of urban dwellers in that study sample (about 5%) with a significant age bias limits inferences about the urban population; that said, activity levels of the three rural tribes^30^ were comparable to those observed in the present study.

The rural–urban gradient in physical activity we observed is likely attributable to differences in labour-intensive occupational activities, more so than leisure-time physical activity (LTPA). In rural communities, with lower socioeconomic status compared to urban areas, many people engage in full-time labour-intensive subsistence farming as a means of livelihood. Apart from social functions such as carnivals which are sporadic, there is little or no notion of LTPA. Therefore, in rural areas, total habitual physical activity is mainly accumulated through occupational activities or other activities of daily living including active transport. This is similar to data from developed countries which show that LTPA is more prevalent in higher socioeconomic strata while the lower socioeconomic classes have a higher proportion of non-LTPA.^31^ ^32^ These differences in non-LTPA among groups of different socioeconomic levels may therefore compromise the validity of questionnaires which collect domain-specific physical activity, particularly those limited to LTPA. Our use of objective continuous monitoring of free-living physical activity overcomes this limitation.

The relative contribution of labour-intensive occupational activity, particularly subsistence farming, to the total habitual physical activity level in rural and urban dwellers can be deduced from the observed seasonal differences of physical activity levels. There was no seasonal variation in activity levels in the urban population in our sample. This may be because occupational activities in urban areas, even when they are labour intensive, are stable throughout the year. Conversely, we observed higher physical activity levels in the rural dwellers during the dry compared to the rainy seasons. The dry seasons correspond to peak subsistence agricultural activities of tilling/planting and harvesting. On the other hand the rainy seasons correspond to the period of waiting for the crops to ripe or the fallow period for the subsequent tilling/planting season.

Physical activity is a complex behaviour which is likely determined by many personal, interpersonal, sociocultural or environmental factors. These factors not only interact with each other, but the interaction may vary by population and by domain or dimension of activity depending on the perception of activity or its correlates.^33^ In this study, the urban dwellers or women generally over-rated their activity level compared to their rural counterparts or men. Also, independent of residential area or sex, more than a third of obese participants perceived their habitual activity as being adequate even though their PAEE and their bout-accumulated MVPA were not different from that of the non-obese participants who said their habitual activity was not adequate. Self-perception of physical activity apparently depends on people's physical or social environment and body size, with a more sedentary environment and being obese leading to an over-perception of physical activity, likely through differences in social norms. Other studies, mainly from developed countries, have reported actual overweight and even self-perception of overweight independent of BMI to be associated with lower physical activity or more sedentary behaviour.^33–35^

Perceptions of the correlates of physical activity may differ between people or populations, but there is unanimity on the health benefits of living physically active lifestyles. It would be expected that education should offer a platform for a better understanding of the importance of physical activity which may influence physical activity behaviour. Most studies, from developed countries, using self-report instruments generally show a positive association between educational level and physical activity.^36–39^ Instead, we observed an inverse association in this study. A higher educational level gives access to more sedentary occupations, leaving the more educated people only leisure time to accumulate activity. In a society where leisure-time activities are not commonplace, the result appears to be a mostly sedentary lifestyle.

In this population, and other low-income countries, it is unclear how health behaviours cluster in individuals. In this study we observed that people with a higher frequency of fruit and vegetable consumption were more physically active in rural and urban areas. However, in resource-limited communities, fruit and vegetable consumption may not be a reflection of deliberate healthy choices but rather a question of accessibility. In a previous study in this population,^40^ rural dwellers had a higher intake of dietary fibre than their urban counterparts, but also had a higher caloric intake and higher alcohol consumption.

This study was cross-sectional and so mainly presents correlates rather than determinants of physical activity. However, it is possible to speculate on the direction of association for some correlates such as urban residence or level of education. For example, it is unlikely that low levels of physical activity would predispose people to acquire more education. The objective measurement of our dependent variables (PAEE and MVPA) precludes observer bias or other differential association with the examined correlates. Despite the precise objective method we used to measure free-living PAEE, the data cannot be subdivided into domains or types of activity. We can only speculate on the relative contributions of different domains of activity to the total PAEE or MVPA, based on the inferences that can be drawn from seasonal differences in the two residential groups.

This is the first study of the differential effects of anthropometric and sociodemographic correlates of objectively measured free-living PAEE and MVPA in rural and urban sub-Saharan Africa. These results highlight some possible effects of urbanisation on patterns and correlates of habitual physical activity in this society which is undergoing socioeconomic and epidemiological transition. These correlates provide information relevant to the development of the content as well as the target of interventions aimed at promoting and maintaining physically active lifestyles in adults in sub-Saharan Africa. Further studies on perceived and actual effects of other correlates such as sociocultural and environmental factors are needed to fully understand the changing patterns and levels of physical activity in this population.

**What is already known on this subject?**

- The health benefits of physical activity are widely known but research on physical activity and its correlates in developing countries such as in sub-Saharan Africa is sparse. The few available studies have mainly used self-reported physical activity, which may be skewed by recall bias. More studies using objective assessment of physical activity and its correlates are needed for the elaboration of appropriate public health interventions to promote physical activity.

**What this study adds?**

- This study provides data on objectively-measured physical activity and some sociodemographic correlates in free-living adults in urban and rural settings in Cameroon. Rural dwellers are significantly more active than urban dwellers; having a higher overall level of physical activity and less sedentary time. In rural and urban dwellers, female gender, obesity and level of education are strongly inversely associated with overall level of physical activity as well as with meeting current physical activity guidelines. Self-perceived adequacy of physical activity level appears to cluster in peers grouped by residence, gender or obesity. These data suggest that urban dwellers, women or obese individuals may benefit more from appropriate and targeted physical activity interventions.

## Supplementary Material

Web figure

Web table
