# Supplementary figures and images for: Patterns and correlates of objectively measured free-living physical activity in adults in rural and urban Cameroon
Source: J Epidemiol Community Health. 2015 Apr 4;69(7):700–7. doi: 10.1136/jech-2014-205154 (PMC4484252; doi:10.1136/jech-2014-205154)

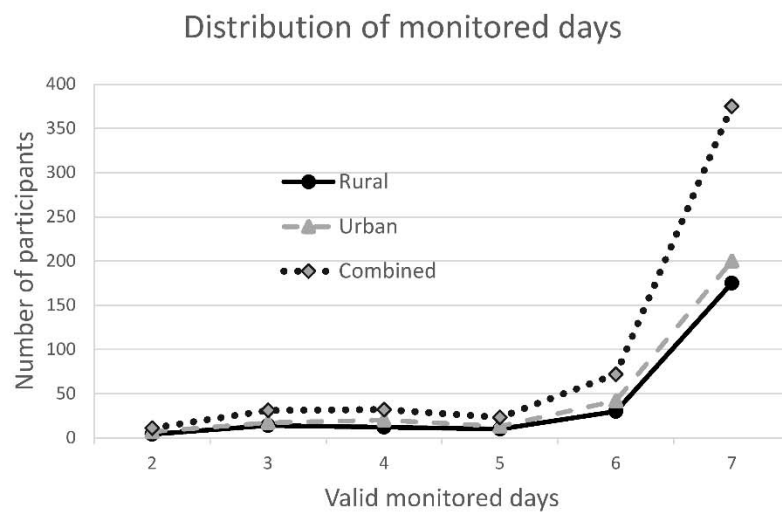

**Suppl Figure 1:** Distribution of valid monitored days between rural and urban dwellers.

Supplement: Web figure [file jech-2014-205154-s1.pdf]
