# Supplementary material for: Patterns and correlates of objectively measured free-living physical activity in adults in rural and urban Cameroon
Source: J Epidemiol Community Health. 2015 Apr 4;69(7):700–7. doi: 10.1136/jech-2014-205154 (PMC4484252; doi:10.1136/jech-2014-205154)
Supplement: Web table [file jech-2014-205154-s2.pdf]

Suppl Table 1: Logistic regression models for socio-personal characteristics of meeting physical activity guidelines in rural and urban adult Cameroonians (N=544).

	OR for meeting PA Guidelines					
	Rural (N=245)			Urban (N=299)		
**Correlates**	**OR**	**95% CI**	**p value**	**OR**	**95% CI**	**p value**
**Anthropometric**						
BMI (kg/m^2)						
Normal	--			--		
Overweight	0.27	0.15, 0.51	<0.001	0.40	0.21, 0.76	0.005
Obese	0.17	0.08, 0.43	<0.001	0.36	0.19, 0.70	0.003
WC (cm)						
Normal	--			--		
Abdominal Obesity	0.48	0.25, 0.90	0.02	0.38	0.20, 0.71	0.002
**Related lifestyle behaviours**						
Smoking						
No	--					
Yes	2.72	0.73, 10.20	0.1	1.02	0.42, 2.50	1
Alcohol drinking						
No	--					
Yes	1.38	0.73, 2.60	0.3	2.32	1.18, 4.53	0.01
Drinking frequency						
<1 day/month	--			--		
1 - 3 days/month	0.93	0.38, 2.32	0.9	1.31	0.60, 2.83	0.5
1 - 4 days/week	1.29	0.49, 3.40	0.6	0.72	0.31, 1.66	0.4
>5 days/week	1.63	0.53, 5.00	0.4	0.45	0.14, 1.41	0.2
Fruits and vegetables						
<3 times/week	--			--		
3 – 6 times/week	2.35	1.23, 4.49	0.01	1.41	0.82, 2.44	0.2
>=7 times/week	3.09	1.40, 6.82	0.005	1.44	0.67, 3.11	0.4
**Social correlates**						
Marital status						
Single	--			--		
Married	1.96	0.95, 4.04	0.07	1.20	0.62, 2.34	0.6
Family size (>15 years)						
1 - 2	--			--		
3 - 5	0.86	0.47, 1.59	0.6	0.79	0.42, 1.50	1
Over 5	0.81	0.36, 1.84	0.6	0.59	0.28, 1.24	0.09
Level of education						
< primary school	--			--		
Primary school	0.65	0.33, 1.29	0.2	0.38	0.16, 0.93	0.03
Secondary school	0.40	0.16, 0.99	0.05	0.32	0.13, 0.72	0.01
University	0.20	0.03, 1.20	0.08	0.26	0.10, 0.69	0.007
Occupational status						
Employed	--			--		
Self-employed	1.84	0.85, 3.98	0.1	1.01	0.51, 1.99	1

Non-paid work	5.33	2.43, 11.70	<0.001	2.32	1.18, 4.59	0.02
Unemployed / retired	1.72	0.57, 5.25	0.3	1.90	0.76, 4.75	0.2
Occupational category						
Light activity	--			--		
Moderate activity	1.62	0.73, 3.59	0.2	0.63	0.31, 1.31	0.2
Intense activity	5.46	2.78, 10.74	<0.001	3.50	1.62, 7.56	0.001

Data are odds ratio for meeting current physical activity guidelines for categories of independent variable, stratified by rural/urban residential site. Models are adjusted for age and sex.
MVPA, moderate-to-vigorous physical activity; BMI, body mass index; WC, waist circumference.
